# Dscam1: Is It a Ubiquitous Code for Dendritic Arborization?

**DOI:** 10.1523/ENEURO.0440-22.2023

**Published:** 2023-01-25

**Authors:** Nikita Kirkise, Katelyn Rygel, Manasi Agrawal

**Affiliations:** 1 Department of Biological Sciences, University of South Carolina, Columbia, South Carolina 29208; 2 School of Biomedical Sciences, Kent State University, Kent, Ohio 44242

Down syndrome cell adhesion molecule (*DSCAM*) is located on human chromosome 21 and triplicated in Down syndrome. *DSCAM* encodes an Ig superfamily cell surface receptor found in both vertebrates and invertebrates. Alternative splicing is more widespread in mammals compared with invertebrates, but *Drosophila Dscam1* is remarkably more complex in this respect than its human counterpart. However, both vertebrate and invertebrate DSCAM/Dscam share a similar critical function—neural wiring (Schmucker and Chen, 2009).

To create both the accuracy and complexity of neural wiring, one of the indispensable characteristics of neurons is the ability to express a wide repertoire of cell surface receptors. This ensures both specificity and selectivity. *Drosophila Dscam1* is one such extraordinary example. *Dscam1* undergoes alternative splicing to generate ∼38,000 receptor isoforms. Dscam1 protein has an intracellular C terminus, a transmembrane domain, and an ectodomain with 10 Ig domains and 6 fibronectin type III repeats. Alternative splicing of the ectodomain region creates ∼19,000 isoforms, each with a unique molecular identity (Schmucker and Chen, 2009).

Interestingly, each neuron expresses ∼5–50 Dscam1 isoforms, giving it a unique code that makes it different from its neighboring neurons (Schmucker and Chen, 2009). This unique code assigned to each neuron helps them distinguish between self and non-self. When neurites with the same Dscam1 isoform interact with each other, it results in homophilic repulsion. Thus, neurites from adjacent neurons interact and form synapses but neurites from the same neuron are evenly spaced, promoting the formation of a specific pattern. Such homophilic interactions between Dscam1 isoforms have been extensively studied for neuronal self-avoidance and tiling—two processes necessary for nervous system development (Hattori et al., 2008).

Dscam1 plays a critical role at multiple stages of neural wiring in *Drosophila*. One of its many functions was first identified in the embryonic fly nervous system. In fly embryos, it is involved in the formation of the CNS axonal tracts. Dscam1 expression in flies is mainly restricted to neural tissue with higher protein levels in axons and dendrites, compared with the soma. *Dscam1*-null flies show defects in CNS axonal patterning and pathfinding at the embryonic stage, and die at the early larval stage (Schmucker and Chen, 2009). In *Drosophila*, mushroom body (MB) neurons form the central structure of the brain that is involved in learning and memory. During development, each axon of a MB neuron splits into two sister axons. These axons exhibit the same isoforms of Dscam1 and, thus, repel each other. This helps the sister branches to grow in separate directions resulting in axonal patterning (Millard and Zipursky, 2008).

Dscam1 also plays a role in the dendritic arborization (da) of neurons. Loss of Dscam1 results in dendrites that cross over and fasciculate, thereby causing targeting errors. In *Drosophila* olfactory interneurons and da neurons, *Dscam1* knockdown leads to dendritic clumping and reduced dendritic branching, respectively (Zipursky and Grueber, 2013). However, there is limited knowledge about the role of Dscam1 in different types of neurons in the CNS.

Dscam1 is abundantly expressed in the developing brain and ventral nerve cord (VNC) during pupal stages 3 through 11, which is when extensive dendritic and axonal growth occurs. However, Dscam1 protein expression decreases in the adult stages. Since Dscam1 appears to be ubiquitously expressed in the brain and VNC, it is plausible that it performs similar functions in all CNS-specific neuronal types. Interestingly, a recent study by Wilhelm et al. (2022) shows a novel role for Dscam1 in the developing CNS of *Drosophila* that is neuronal specific and developmental stage specific. The authors examined the function of Dscam1 in central neuron dendrite and axon development by knocking down Dscam1 during the development of both larval and adult efferent neurons and in five types of interneurons (Fig. 1; Wilhelm et al., 2022).

**Figure 1.**
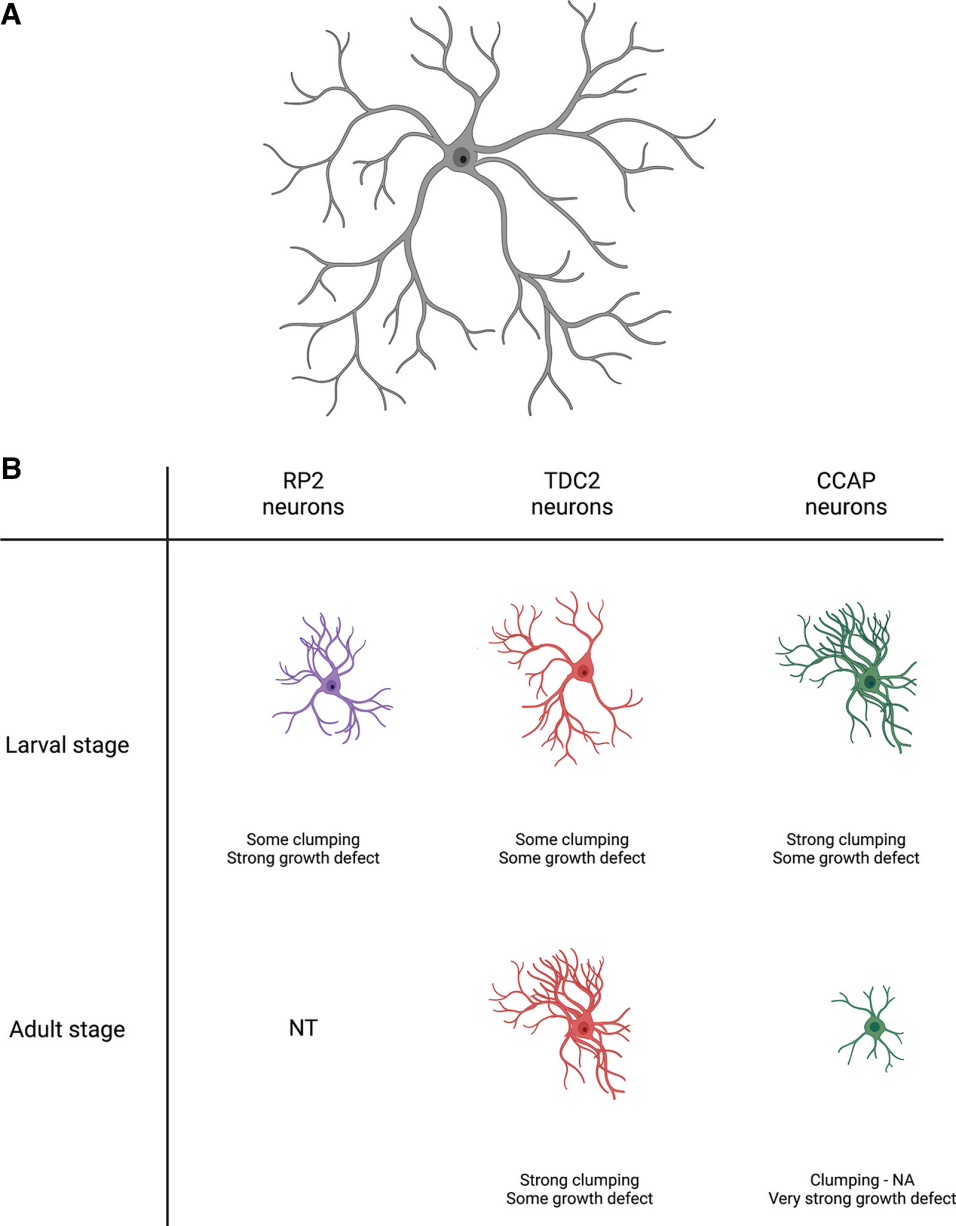
Summary of the effects of *Dscam1* knockdown on dendritic clumping and dendritic growth in both larval and adult efferent neurons. ***A***, Representative image of typical self-avoidance in neurons. ***B***, Overview of dendritic clumping and growth defect found in each efferent neuron type at both larval and adult stages following *Dscam1* knockdown. RP2 neurons showed some clumping and a strong growth defect in the larval stage, but the adult stage was not tested (NT). TDC2 neurons showed some growth defect and clumping in both stages, with stronger clumping seen in the adult stage. CCAP neurons had strong clumping and some growth defect in the larval stage but at the adult stage, dendritic clumping could not be analyzed (NA) because of a very strong growth defect following *Dscam1* knockdown. All interneurons examined (data not shown) did not show a defect in dendritic self-avoidance or growth. Axons are not shown for simplification because axonal arborization showed no defect in self-avoidance or growth following *Dscam1* knockdown.

*Dscam1* knockdown in larval RP2 motoneurons and larval tyrosine decarboxylase (TDC2) aminergic neurons results in dendritic clumping. Furthermore, this dendritic clumping is much higher in adult TDC2 aminergic neurons and in larval crustacean cardioactive peptide (CCAP) peptidergic neurons (Fig. 1). In addition to the defects in dendritic self-avoidance following *Dscam1* knockdown, all efferent neurons show impaired dendritic growth. However, this impairment is stronger in the larval RP2 motoneurons and the adult CCAP peptidergic neurons. Interestingly, *Dscam1* knockdown had no effect on either dendritic self-avoidance or growth in any type of interneuron examined, including contralateral serotonergic deuterocerebral, lobular columnar type 4, period, basin, and giant fiber interneurons. This suggests that Dscam1 has no role in dendritic development in these interneurons.

Previous studies have reported that *Dscam1* knockdown hinders the collateral formation of mechanosensory axons in *Drosophila* (He et al., 2014). Contrary to this, in the present study, the authors did not observe any changes in axonal growth or axonal self-avoidance following *Dscam1* knockdown in any of the neuronal types tested, including mushroom body neurons. Overall, the current study shows that Dscam1 has differential effects in *Drosophila* efferent neurons and interneurons.

This study thus highlights that the role of Dscam1 in dendritic self-avoidance and growth is not ubiquitous among all central neurons. Whether impaired self-avoidance leads to dendritic growth defects or vice versa still remains elusive. However, dendritic self-avoidance in interneurons appears to be regulated by a mechanism that is entirely independent of Dscam1. It is also noteworthy that Dscam1, which is found in both axons and dendrites, plays a role in dendritic, but not axonal, arborization in these central neurons. The findings of this study thus raise the following questions. (1) What role does Dscam1 play in the axons of these central neurons? (2) How do interneurons regulate dendritic spacing? (3) Is there a mechanism that compensates for loss of Dscam1 in interneurons? (4) Why are there differences in the temporal functions of Dscam1 during development in the various cell types? (5) What are the signaling pathways through which Dscam1 regulates dendritic patterning in these neurons? Answering these questions will provide greater insights into how Dscam1 regulates neural wiring.

## Synthesis

Reviewing Editor: Jeffery Twiss, University of South Carolina

## References

[B1] Hattori D, Millard SS, Wojtowicz WM, Zipursky SL (2008) Dscam-mediated cell recognition regulates neural circuit formation. Annu Rev Cell Dev Biol 24:597–620. 10.1146/annurev.cellbio.24.110707.175250 18837673 PMC2711549

[B2] He H, Kise Y, Izadifar A, Urwyler O, Ayaz D, Parthasarthy A, Yan B, Erfurth M-L, Dascenco D, Schmucker D (2014) Cell-intrinsic requirement of Dscam1 isoform diversity for axon collateral formation. Science 344:1182–1186. 10.1126/science.1251852 24831526

[B3] Millard SS, Zipursky SL (2008) Dscam-mediated repulsion controls tiling and self-avoidance. Curr Opin Neurobiol 18:84–89. 10.1016/j.conb.2008.05.005 18538559 PMC2707353

[B4] Schmucker D, Chen B (2009) Dscam and DSCAM: complex genes in simple animals, complex animals yet simple genes. Genes Dev 23:147–156. 10.1101/gad.1752909 19171779

[B5] Wilhelm N, Kumari S, Krick N, Rickert C, Duch C (2022) Dscam1 has diverse neuron type-specific functions in the developing *Drosophila* CNS. eNeuro 9:ENEURO.0255-22.2022. 10.1523/ENEURO.0255-22.2022 PMC9417601 35981870

[B6] Zipursky SL, Grueber WB (2013) The molecular basis of self-avoidance. Annu Rev Neurosci 36:547–568. 10.1146/annurev-neuro-062111-150414 23841842

